# Use of ITS2 Region as the Universal DNA Barcode for Plants and Animals

**DOI:** 10.1371/journal.pone.0013102

**Published:** 2010-10-01

**Authors:** Hui Yao, Jingyuan Song, Chang Liu, Kun Luo, Jianping Han, Ying Li, Xiaohui Pang, Hongxi Xu, Yingjie Zhu, Peigen Xiao, Shilin Chen

**Affiliations:** 1 Institute of Medicinal Plant Development, Chinese Academy of Medical Sciences, Peking Union Medical College, Beijing, People's Republic of China; 2 College of Pharmacy, Hubei University of Chinese Medicine, Wuhan, Hubei, People's Republic of China; 3 School of Bioscience and Engineering, Southwest Jiaotong University, Chengdu, Sichuan, People's Republic of China; 4 Chinese Medicine Laboratory, Hong Kong Jockey Club Institute of Chinese Medicine, Hong Kong, People's Republic of China; Lund University, Sweden

## Abstract

**Background:**

The internal transcribed spacer 2 (ITS2) region of nuclear ribosomal DNA is regarded as one of the candidate DNA barcodes because it possesses a number of valuable characteristics, such as the availability of conserved regions for designing universal primers, the ease of its amplification, and sufficient variability to distinguish even closely related species. However, a general analysis of its ability to discriminate species in a comprehensive sample set is lacking.

**Methodology/Principal Findings:**

In the current study, 50,790 plant and 12,221 animal ITS2 sequences downloaded from GenBank were evaluated according to sequence length, GC content, intra- and inter-specific divergence, and efficiency of identification. The results show that the inter-specific divergence of congeneric species in plants and animals was greater than its corresponding intra-specific variations. The success rates for using the ITS2 region to identify dicotyledons, monocotyledons, gymnosperms, ferns, mosses, and animals were 76.1%, 74.2%, 67.1%, 88.1%, 77.4%, and 91.7% at the species level, respectively. The ITS2 region unveiled a different ability to identify closely related species within different families and genera. The secondary structure of the ITS2 region could provide useful information for species identification and could be considered as a molecular morphological characteristic.

**Conclusions/Significance:**

As one of the most popular phylogenetic markers for eukaryota, we propose that the ITS2 locus should be used as a universal DNA barcode for identifying plant species and as a complementary locus for CO1 to identify animal species. We have also developed a web application to facilitate ITS2-based cross-kingdom species identification (http://its2-plantidit.dnsalias.org).

## Introduction

As one of the most important markers in molecular systematics and evolution [1]–[6], ITS2 shows significant sequence variability at the species level or lower. The availability of its structural information permits analysis at higher taxonomic level [1], [3], [7]–[9], which provides additional information for improving accuracy and robustness in the reconstruction of phylogenetic trees [10]. Furthermore, ITS2 is potentially useful as a standard DNA barcode to identify medicinal plants [11]–[15] and as a barcode to identify animals [16]–[19]. ITS2 is regarded as one of the candidate DNA barcodes because of its valuable characteristics, including the availability of conserved regions for designing universal primers, the ease of its amplification, and enough variability to distinguish even closely related species.

Since Hebert first proposed the use of the cytochrome *c* oxidase subunit 1 (CO1) as a barcode to identify animals, DNA barcoding has attracted worldwide attention [20], [21]. Many loci have been proposed as plant barcodes, including ITS [22], [23], *rbcL* [24], [25], *psbA-trnH* [24], [26], [27], and *matK* [26]–[28]. Most recently, the Plant Working Group of the Consortium for the Barcode of Life recommended a two-locus combination of *rbcL* + *matK* as a plant barcode [29]. However, some researchers have suggested that DNA barcodes based on uniparentally inherited markers can never reflect the complexity that exists in nature [22]. In addition, nuclear genes can provide more information than barcoding based on organellar DNA, which is inherited from only one parent [30].

Although ITS2 shows a great potential as a barcode to identify plants and animals, an extensive evaluation based on a comprehensive sample set is lacking. To validate the potential of using the ITS2 region to identify closely related species of plants and animals, we analyzed 50,790 plant and 12,221 animal ITS2 sequences (Table S1) available in a public database. The results support the conclusion that the ITS2 region can be used as an effective barcode for the identification of plant species and as a complementary locus to CO1 for identifying animals.

## Results

**Figure 1:**
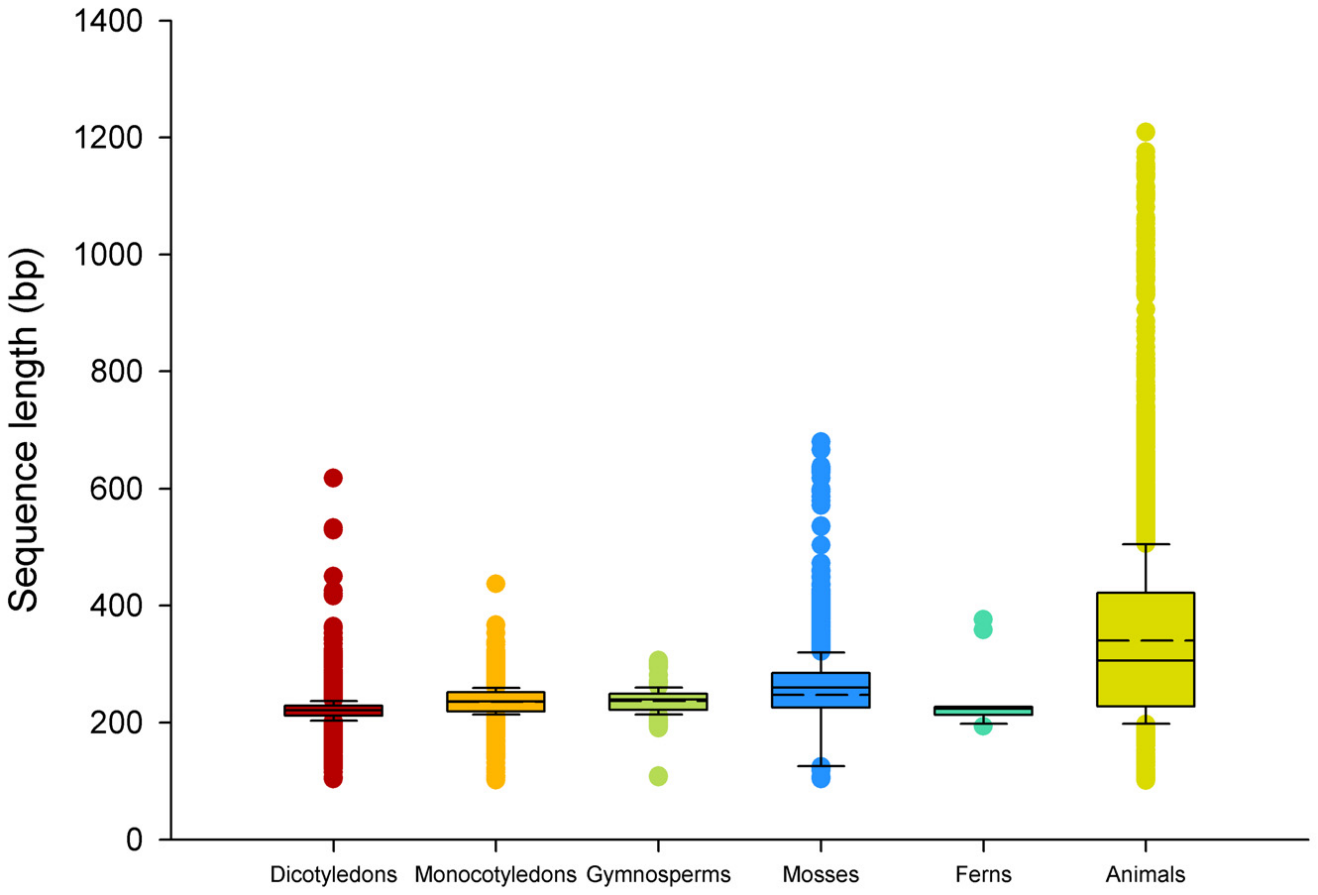
Box plots of the ITS2 sequence length of plants and animals. In a box plot, the box shows the interquartile range (IQR) of the data. The IQR is defined as the difference between the 75th percentile and the 25th percentile. The solid and dotted line through the box represent the median and the average length, respectively.

**Figure 2:**
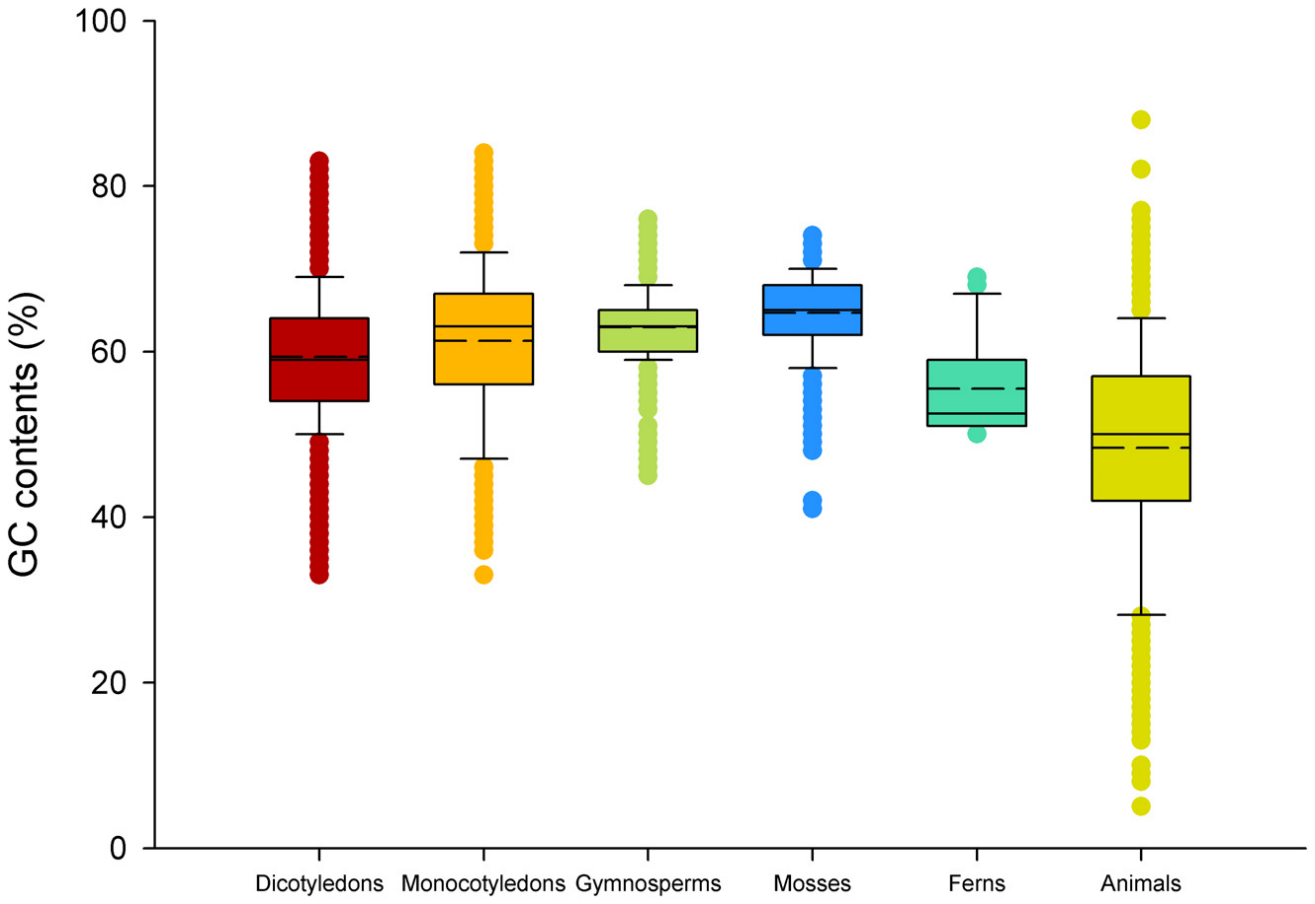
Box plots of GC contents of ITS2 of plants and animals. In a box plot, the box shows the IQR of the data. The IQR is defined as the difference between the 75th percentile and the 25th percentile. The solid and dotted line through the box represent the median and the average GC contents, respectively.

For plants, the lengths of ITS2 sequences from dicotyledons and mosses were distributed between 100 and 700 bp, and the lengths of ITS2 sequences from monocotyledons, gymnosperms, and ferns were distributed between 100 and 480 bp. The average lengths of ITS2 sequences for dicotyledons, monocotyledons, gymnosperms, ferns, and mosses were 221, 236, 240, 224, and 260 bp, respectively. For animals, the ITS2 sequence lengths ranged from 100 to 1,209 bp (mainly dispersed between 195 and 510 bp), with an average of 306 bp. The GC contents of the ITS2 sequences of the dicotyledons, monocotyledons, gymnosperms, ferns, mosses, and animals were calculated, and the averages were 59.4%, 61.3%, 62.9%, 55.5%, 64.7%, and 48.3%, respectively. The average and distributions of ITS2 sequence lengths, as well as the GC contents of the six taxa, are shown in Figure 1 and Figure 2, respectively.

Inter-specific divergence was assessed by three parameters: average inter-specific distance, average theta prime, and smallest inter-specific distance [11], [31], [32]. In contrast, intra-specific variation was evaluated by three additional parameters: average intra-specific difference, theta (θ), and average coalescent depth [27], [32]. The inter-specific genetic distances between congeneric species of plants and animals were greater than the intra-specific variations of the ITS2 regions of the different taxa (Table 1).

**Table 1:** Analysis of intra- and inter-specific divergences of congeneric species in plants and animals.

Taxa	Animals	Dicotyledons	Monocotyledons	Gymnosperms	Mosses	Ferns
**All inter-specific distance**	0.3761±0.5982	0.1042±0.1393	0.1829±0.1940	0.0537±0.0892	0.1007±0.0913	0.4758±0.3547
**Theta prime**	0.2820±0.4257	0.0999±0.1118	0.1127±0.1310	0.0573±0.0744	0.1874±0.1792	0.4995±0.2906
**Minimum inter-specific distance**	0.1361±0.2254	0.0370±0.0667	0.0386±0.0809	0.0195±0.0576	0.0838±0.1466	0.2399±0.3173
**All intra-specific distance**	0.0522±0.1150	0.0214±0.0809	0.0309±0.0712	0.0170±0.0413	0.0114±0.0456	0.0082±0.0160
**Theta**	0.0274±0.0809	0.0231±0.0781	0.0244±0.0764	0.0255±0.0511	0.0289±0.0792	0.0262±0.0254
**Coalescent depth**	0.0596±0.1962	0.0363±0.1739	0.0360±0.1213	0.0368±0.0653	0.0452±0.1087	0.0336±0.0256

BLAST1 method based on similarity was used to evaluate the identification capacity of the ITS2 region [33]. At the genus level, the use of the ITS2 region had a >97% success rate for the identification of plants and animals (Table 2). At the species level, ITS2 sequences correctly identified 91.9% of 12,221 animal samples, whereas the success rates of using ITS2 sequences for the identification of 34,676 dicotyledons, 11,598 monocotyledons, 946 gymnosperms, 42 ferns, and 3,528 mosses were 76.1%, 74.2%, 67.1%, 88.1%, and 77.4% at the species levels, respectively (Table 2).

**Table 2:** Identification efficiency of ITS2 regions in plants and animals using BLAST1 method.

Taxa	Taxa level	Correct identification (%)	Ambiguous identification (%)
Animals	Species	91.7	8.3
	Genus	99.7	0.3
Dicotyledons	Species	76.1	23.9
	Genus	99.1	0.9
Monocotyledons	Species	74.2	25.8
	Genus	97.9	2.1
Gymnosperms	Species	67.1	32.9
	Genus	99.5	0.5
Mosses	Species	77.4	22.6
	Genus	98.6	1.4
Ferns	Species	88.1	11.9
	Genus	100.0	0

**Figure 3:**
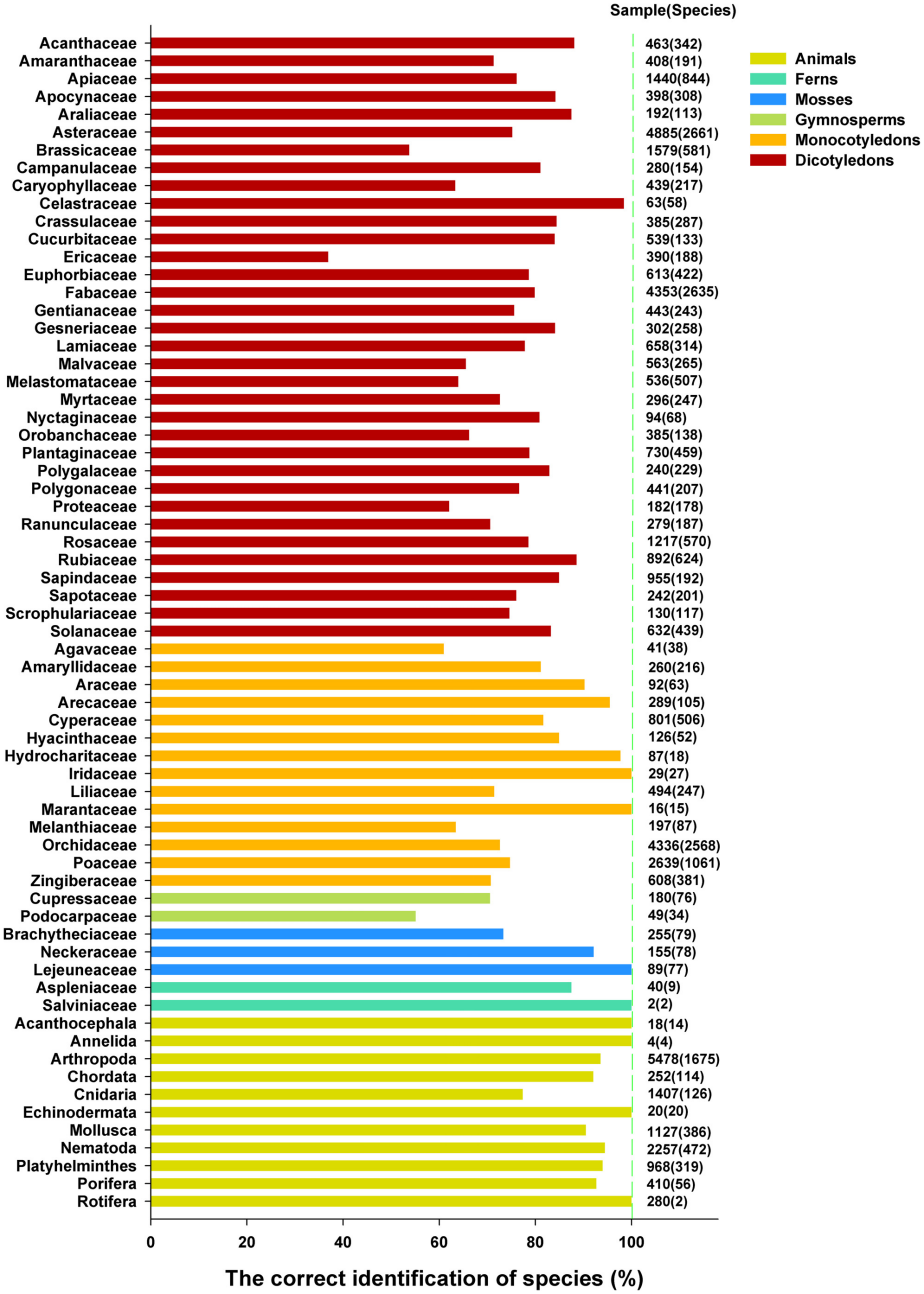
Identification efficiency when using ITS2 regions to distinguish between closely related species in different families of plants and animals using the BLAST1 method. The ITS2 sequences of all animal phyla, dicotyledon, gymnosperm, and mosses families with more than 10 genera, monocotyledon families with more than 5 genera, and all fern families are shown in this figure.

In addition, we studied the possibility of using ITS2 sequences to identify closely related species in different families. First, we studied 34 dicotyledon families, each having more than 10 genera. For 13 families, the rates of successful identification were more than 80%; success rates for identification fell below 70% in only seven families (Fig. 3). Of the 14 monocotyledon families that each had more than 5 genera, identification success rates were lower than 70% in only two families (Fig. 3). The success rates for using the ITS2 region to identify species in families with more than 10 genera of mosses and gymnosperms and all families of ferns are also shown in Fig. 3. The success rates for using the ITS2 region to identify species in families with less than 10 genera of dicotyledons, mosses, gymnosperms, and with less than 5 genera of monocotyledons are listed in Table S2. Compared to the success rates when identifying species in plants, the success rates for identifying species in the nine phyla of animals studied were much higher (more than 90%), except for Cnidaria (77.1%) (Fig. 3).

Second, we focused on the ability of ITS2 to discriminate amongst the lower taxa. Of the 35 dicotyledon genera that each had more than 80 species, identification success rates were more than 80% for 12 genera. The success rates for identification of species within the *Draba* and *Rhododendron* genera were the two lowest at 27.2% and 21.9%, respectively (Table 3). The success rates for the identification of species within the dicotyledon genera with less than 80 species can be found in Table S3. Of the 42 monocotyledon genera with more than 30 species, identification success rates were greater than 80% in 13 genera. The success rates for identification of species within the *Kniphofia*, *Ophrys*, and *Diuris* genera were the three lowest at 16.2%, 22.7%. and 31.1%, respectively (Table 4). The success rates for the identification of species within genera with less than 30 species of monocotyledons and of species from different genera of gynosperms, ferns, and mosses can be found in Table S3. All 28 animal genera with more than 20 species each had a species identification success rates greater than 80%, except for the genus *Calligrapha* and *Dolichopus*. The success rates for the identification of species within the genus *Calligrapha* and *Dolichopus* were the lowest, which were at 73.3% and 73.8%, respectively (Table 5). The success rates for the identification of genera with less than 20 species of animals are presented in Table S3.

**Table 3:** Success rates of ITS2 for species identification in genera with more than 80 species in dicotyledons.

Family name	Genus name	No. of species	No. of samples	Success rate at the species level (%)
Fabaceae	*Astragalus*	322	381	65.9
Fabaceae	*Indigofera*	234	266	95.5
Fabaceae	*Trifolium*	223	334	70.1
Melastomataceae	*Miconia*	206	223	66.4
Brassicaceae	*Draba*	199	452	27.2
Asteraceae	*Centaurea*	185	284	58.5
Plantaginaceae	*Veronica*	178	264	90.2
Oxalidaceae	*Oxalis*	176	201	80.6
Moraceae	*Ficus*	174	215	85.6
Solanaceae	*Solanum*	162	248	83.9
Asteraceae	*Senecio*	161	219	77.6
Fabaceae	*Aspalathus*	138	165	55.8
Fabaceae	*Acacia*	127	151	72.8
Rosaceae	*Rubus*	124	199	72.9
Begoniaceae	*Begonia*	124	236	97.9
Polygalaceae	*Polygala*	123	128	89.8
Asteraceae	*Artemisia*	118	159	63.5
Rosaceae	*Cliffortia*	118	151	67.5
Acanthaceae	*Ruellia*	117	151	79.5
Euphorbiaceae	*Euphorbia*	117	168	86.9
Balsaminaceae	*Impatiens*	117	137	97.8
Apiaceae	*Eryngium*	113	136	62.5
Myrtaceae	*Eucalyptus*	106	135	61.5
Euphorbiaceae	*Croton*	104	142	59.9
Calceolariaceae	*Calceolaria*	99	103	74.8
Convolvulaceae	*Cuscuta*	98	261	74.7
Caryophyllaceae	*Dianthus*	97	141	40.4
Lamiaceae	*Salvia*	96	213	81.2
Berberidaceae	*Berberis*	94	164	55.5
Ericaceae	*Rhododendron*	86	233	21.9
Euphorbiaceae	*Macaranga*	84	127	66.9
Sapindaceae	*Acer*	83	745	81.5
Rosaceae	*Prunus*	82	222	78.8
Urticaceae	*Pilea*	81	88	97.7
Rubiaceae	*Coffea*	81	111	72.1

**Table 4:** Success rates of ITS2 for species identification in genera with more than 30 species in monocotyledons.

Family name	Genus name	No. of species	No. of samples	Success rate at the species level (%)
Alliaceae	*Allium*	273	717	72.7
Amaryllidaceae	*Cyrtanthus*	43	57	86.0
Amaryllidaceae	*Crinum*	34	34	52.9
Arecaceae	*Pinanga*	49	161	95.7
Asphodelaceae	*Kniphofia*	52	99	16.2
Costaceae	*Costus*	50	94	52.1
Cyperaceae	*Carex*	318	506	80.6
Cyperaceae	*Eleocharis*	52	122	90.2
Hyacinthaceae	*Lachenalia*	31	50	70.0
Juncaceae	*Luzula*	45	56	51.8
Juncaceae	*Juncus*	42	51	68.6
Liliaceae	*Gagea*	79	228	56.1
Liliaceae	*Lilium*	78	124	79.0
Liliaceae	*Fritillaria*	49	58	82.8
Musaceae	*Musa*	37	63	82.5
Orchidaceae	*Maxillaria*	227	482	62.9
Orchidaceae	*Oncidium*	139	215	65.1
Orchidaceae	*Dendrobium*	121	160	91.9
Orchidaceae	*Disa*	120	143	79.7
Orchidaceae	*Ophrys*	100	260	22.7
Orchidaceae	*Paphiopedilum*	85	192	76.6
Orchidaceae	*Phalaenopsis*	56	232	65.9
Orchidaceae	*Masdevallia*	48	49	79.6
Orchidaceae	*Gomesa*	46	55	49.1
Orchidaceae	*Satyrium*	42	59	98.3
Orchidaceae	*Dendrochilum*	42	52	71.2
Orchidaceae	*Cyrtochilum*	41	75	69.3
Orchidaceae	*Telipogon*	38	46	76.1
Orchidaceae	*Dichaea*	36	66	81.8
Orchidaceae	*Diuris*	33	61	31.1
Orchidaceae	*Scaphyglottis*	33	40	100.0
Orchidaceae	*Cymbidium*	30	58	74.1
Poaceae	*Poa*	115	178	46.1
Poaceae	*Bromus*	66	80	76.3
Poaceae	*Elymus*	54	155	74.2
Poaceae	*Festuca*	51	69	72.5
Poaceae	*Nassella*	31	36	80.6
Poaceae	*Hordeum*	31	481	81.7
Potamogetonaceae	*Potamogeton*	33	211	72.5
Zingiberaceae	*Globba*	60	103	57.3
Zingiberaceae	*Alpinia*	46	85	68.2
Zingiberaceae	*Amomum*	37	52	94.2

**Table 5:** Success rates of ITS2 for species identification in genera with more than 20 species in animals.

Family name	Genus name	No. of species	No. of samples	Success rate at the species level (%)
Aphelenchoididae	*Bursaphelenchus*	32	86	81.4
Camaenidae	*Satsuma*	27	122	100.0
Ceratopogonidae	*Culicoides*	39	134	100.0
Chrysomelidae	*Timarcha*	42	183	97.3
Chrysomelidae	*Calligrapha*	23	45	73.3
Clausiliidae	*Albinaria*	25	31	96.8
Clausiliidae	*Isabellaria*	20	23	95.7
Conidae	*Conus*	23	23	100.0
Culicidae	*Culex*	23	241	98.8
Culicidae	*Aedes*	21	154	93.5
Dolichopodidae	*Dolichopus*	38	65	73.8
Drosophilidae	*Drosophila*	40	43	81.4
Enidae	*Mastus*	24	44	95.5
Gyrodactylidae	*Gyrodactylus*	49	135	99.3
Heteroderidae	*Heterodera*	41	211	93.8
Longidoridae	*Xiphinema*	25	52	100.0
Lycaenidae	*Agrodiaetus*	75	111	90.1
Nesticidae	*Nesticus*	26	51	100.0
Nitidulidae	*Meligethes*	79	82	87.8
Planorbidae	*Biomphalaria*	22	91	95.6
Poritidae	*Porites*	20	206	89.3
Pratylenchidae	*Pratylenchus*	22	154	97.4
Psychodidae	*Phlebotomus*	24	129	100.0
Reduviidae	*Triatoma*	28	127	94.5
Sarcophagidae	*Sarcophaga*	24	33	100.0
Simuliidae	*Simulium*	22	177	80.8
Steinernematidae	*Steinernema*	46	140	96.4
Trichogrammatidae	*Trichogramma*	59	278	99.3

**Figure 4:**
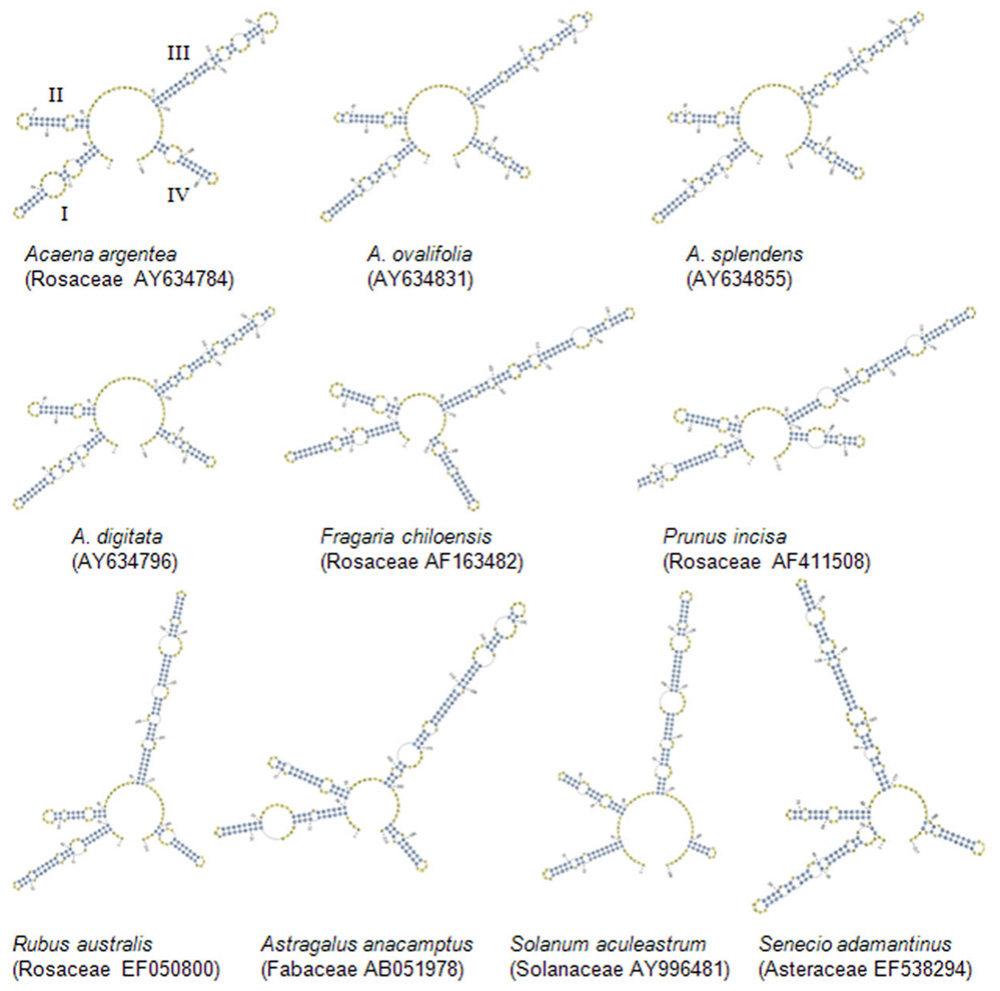
The secondary structure of ITS2 in different species of dicotyledons.

To identify the species, we focused not only on the divergence of primary sequences of ITS2, but also on the use of variations in the secondary structures of ITS2. The secondary structures and alignments of primary sequences of ITS2 were reconstructed in four different species from the same genus, four species from different genera of the same family, and four species from the different families of dicotyledons, monocotyledons, and animals. These are shown in Figures 4, S1, S2, S3, S4, and S5. All of the secondary structures in these species have four similar helices: Helix I, II, III, and IV (Figs. 4, S2 and S4) [2], [34], [35]. Helix III is relatively longer than the others. At the different taxa levels of dicotyledons, monocotyledons, and animals, the secondary structures show different levels of similarity, which result from the differences in the primary sequences of these species. Thus, the species of dicotyledons, monocotyledons, and animals could be identified by their secondary structure. And, the secondary structure of the ITS2 region could be considered as a molecular morphological characteristic.

Although ITS2 sequences are advantageous for identification purposes, one of the concerns for accepting the ITS2 region as a barcode is the potential contamination of fungal sequences [11]. We checked the studied ITS2 sequences of plants and animals using the Hidden Markov model (HMM) for fungal ITS2 annotation, in addition to conducting BLAST searches of the fungal nrITS database [36]. For the plants, 139 and 136 ITS2 sequences may have been fungal sequences, as determined by BLAST and HMM, respectively. Less than 10 ITS2 sequences of gynosperms, ferns, and mosses may have been fungal sequences, as determined by the BLAST and HMM. There were 37 and 32 dicotyledon ITS2 sequences, as well as 30 and 27 animal ITS2 sequences that may have been fungal sequences as determined by the BLAST and HMM, respectively. There were 86 monocotyledon ITS2 sequences that may have been fungal sequences (Table S4).

Finally, we developed a web application at http://its2-plantidit.dnsalias.org to allow researchers to further test the usefulness of ITS2 for species identification across plant and animal kingdoms. Four different modules have been implemented at the time of this writing. The first module, “View,” provides a gene-card like summary regarding the ITS2 reference sequence for a particular species. The users perform a query with a taxonomy ID used in NCBI's taxonomy browser. The module then displays all sequences associated with the taxonomy ID, as well as the reference barcode sequences for the ITS2 region of this species. The second module, “Retrieve,” allows the user to retrieve various segments of the ITS2 region, which can be divided into the 5.8S gene segment, the ITS2 core region, and the 28S gene segment. The sequences for these different regions can then be used to build various models, such as HMMs. The third module, “Annotate,” allows users to annotate the 5.8S gene segment, the ITS2 core region, and the 28S gene segment for their own sequences. The users need to provide the alignment of multiple sequences for the 5.8S gene and the 28S gene segments. The module then builds HMMs with these fragments, and uses HMM to query the input sequences to define the boundaries of the various fragments. The users can choose to export various segments individually or by batch. The last module, “Identify,” performs a BLAST search on a query sequence against our internal ITS2 reference barcode sequence database. Species identification is based on the assumption that the ITS2 sequence for this species is included in the reference database. In such a case, if the top hit represents a unique species, this species should represent the species to which the sample belongs. In contrast, if the top hit includes more than one unique species, the ITS2 sequence cannot be used to identify the sample, and additional DNA barcodes are needed to resolve the identity of the sample. If the reference database does not contain the ITS2 sequence of the species under investigation, the identification is more complicated, and has been stated elsewhere [33].

In summary, a comprehensive reference database is critical for species identification, which is the reason this database was constructed.

## Discussion

An ideal barcode should possess sufficient variation among the sequences to discriminate species; however, it also needs to be sufficiently conserved so that there is less variability within species than between species [37], [38]. Chen et al. (2010) compared seven candidate DNA barcodes (*psbA-trnH, matK, rbcL, rpoC1, ycf5*, ITS2, and ITS) from medicinal plant species and proposed that ITS2 can be potentially used as a standard DNA barcode to identify medicinal plants. The ITS2 region has also been used as a barcode to identify spider mites [41], *Sycophila* [16], and *Fasciola* [18]. In the present study, we extended this analysis across all plants and animals, and assessed the species discrimination capacity of ITS2 sequences for 50,790 plant and 12,221 animal sequences (Table S1). The success rates for identification of plants and animals were more than 97% and 74% at the genus and species level (Table 2), respectively, except for gymnosperms, which had a 67.1% success rate at the species level. In addition, the ITS2 region had a high success rate for discriminating between closely related species in plants and animals (Fig. 3, Tables 3, 4, 5, S2, and S3). The sequence length of ITS2 is short (Fig. 1), which satisfies the requirements for PCR amplification and sequencing. Finally, the secondary structures of ITS2 are conserved and can provide useful biological information for alignment [2], [4], [35]; thus, it can be considered as molecular morphological characteristics for species identification.

The ITS2 sequence lengths of plants and animals were mainly distributed in the 195–510 bp range. The identification of plant and animal voucher species and other collections using DNA barcoding techniques is one of the main tasks in natural museums and research institutes. The length of the ITS2 region is sufficiently short to allow amplification of even degraded DNA. In addition, the intra-specific variations in plants and animals are lower than the inter-specific divergences. But the overlap of genetic variation without barcoding gaps significantly increases when the number of closely related species is increased [32].

Hebert et al. found that more than 98% of 13,320 congeneric species pairs, including representatives from 11 phyla, have sufficient sequence divergence to ensure easy identification [20]. However, the sequence divergence of COI for some animal species, such as *cnidarians* [20] and the West Palaearctic *Pandasyopthalmus* taxa [39], is relatively low, and even invariant. In addition, mtDNA is maternally inherited; other resources of data should be considered, such as nuclear DNA, morphology, or ecology [40]. The success rate of using ITS2 for identification of animals is 91.7% at the species level based on testing of a comprehensive sample set, and the identification efficiency of ITS2 for sequences in *cnidarians* is more than 77%. ITS2 sequences have a relatively high divergence rate; thus, it can be used as a complementary locus to CO1 for identification of animal species.

Recently, ITS2 region has been found to vary in primary sequences and secondary structures in a way that correlates highly with taxonomic classification. Several researchers have already demonstrated the potential for using ITS2 for taxonomic classification and phylogenetic reconstruction at both the genus and species levels for eukaryotes, including animals, plants, and fungi [2], [4], [8], [9], [42], [43]. The ITS2 region of nuclear DNA provides a powerful tool because of sufficient variation in primary sequences and secondary structures. Analysis of the secondary structures formed by the RNA transcript as it folds back upon itself at transcription has been less commonly conducted; however, it has been proven extremely useful in aiding proper sequence alignment [1], [44]. Schultz and Wolf described the utilization of ITS2′s primary sequence and secondary structure information, together with an ITS2-specific scoring matrix and an ITS2-specific substitution model, based on tools such as 4SALE, the CBCAnalyzer, and ProfDistS [9].

Among of 50,790 ITS2 sequences of plants and 12,221 ITS2 sequences of animals,139 and 30 sequences, respectively, could be fungal sequences. Thus, the frequency is less than 0.3% in both plants and animals. This result is similar to that of Chen et al. [11]. The frequency of suspected fungal sequences in monocotyledon ITS2 sequences is twice as high as in dicotyledons, which may be due to the presence of endophytic fungi in most monocotyledon species. Although the rate of fungal contamination is very low, we should pay more attention to the data from the public database [11].

There are multiple copies of ITS (containing ITS1 and ITS2) in plants and animals. Although different copies of ITS exist, which may result in misleading phylogenetic inferences [45], there remain several advantages for its widespread use, such as the levels of variations and multicopy structure facilitating PCR amplification, even from herbarium specimens [46].

In conclusion, we believe that the ITS2 locus can be used as a barcode for authenticating plant species, as well as a complementary locus to CO1 for identifying animal species. The sequences of the universal primers and the amplification conditions for obtaining the ITS2 sequences of plants and animals can be found in Table S5, as well as in the ITS2 application web. There were limited ITS2 sequences of ferns and vertebrates in the GenBank; therefore, the success rates for ITS2 to identify them need further investigation.

## Materials and Methods

### Reference Database Construction

**Figure 5:**
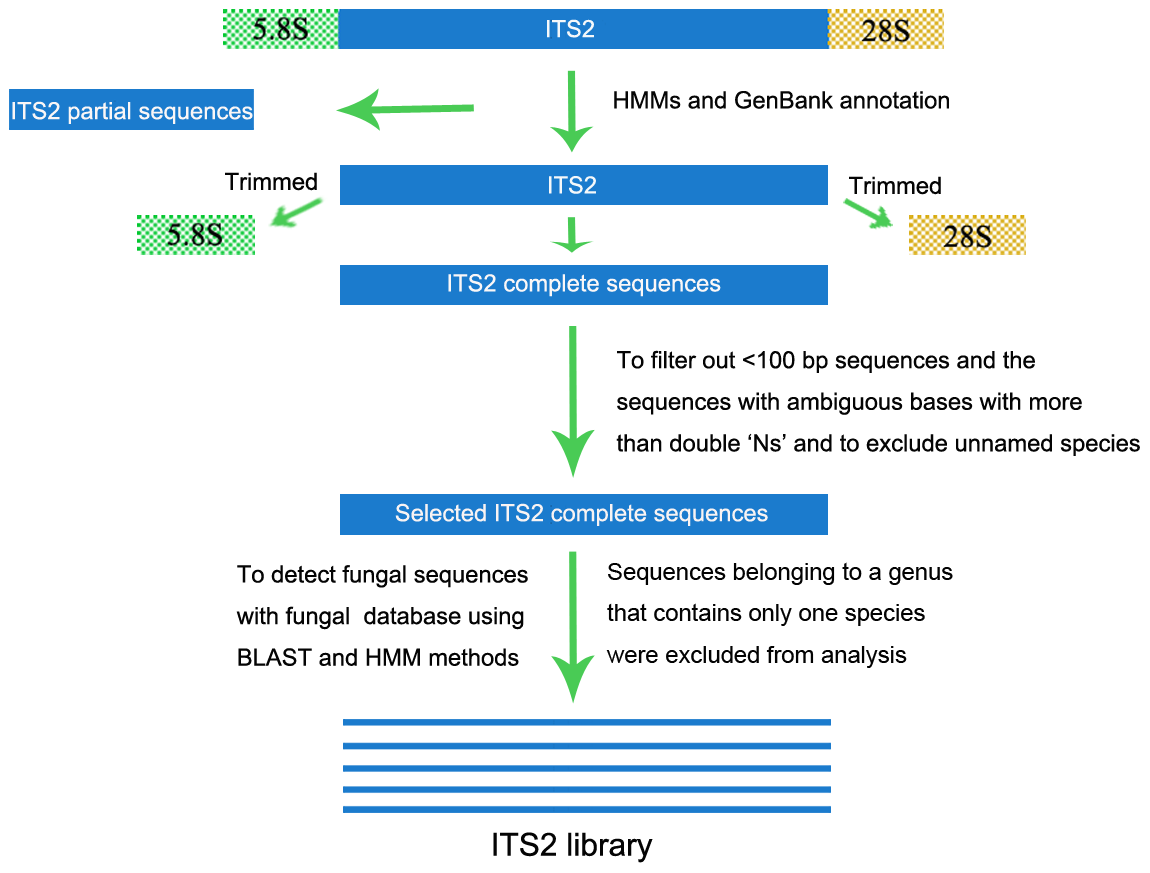
The workflow diagram for the construction of ITS2 sequences libraries.

All ITS2 sequences of dicotyledons, monocotyledons, gymnosperms, mosses, ferns and animals were downloaded from GenBank on June 28, 2010 by searching using the keywords “internal transcribed spacer 2,” which retrieved 160,295 sequences. These sequences were used to construct an analysis dataset. The raw data were annotated and trimmed using ITS2 annotation tools based on HMM [42]. Two conserved regions of the 5.8S and 28S gene for plants and animals, respectively, were used to delimit the ITS2 region. A maximum E-value of 1.0 was used. The trimmed sequences were edited manually. The sequences with less than 100 bp length, or with ambiguous bases with more than two “Ns”, or with unnamed species (such as those with spp. and aff. in the species name) were excluded. The selected ITS2 sequences were filtered then with a HMM-based annotation [35] and fungal nrITS database (http://www.emerencia.org/fungalitspipeline.html) [36] using the BLAST tool. The ITS2 sequences belonging to a genus that contains only one species were excluded from the analysis. Finally, a reference database was constructed. The detailed sequences information can be found in Table S6. The workflow is shown in Figure 5.

### GC Content, Sequence Length, and Intra- and Inter-specific Divergence

The GC content and sequence length were calculated for all of the ITS2 sequences of dicotyledons, monocotyledons, gymnosperms, ferns, mosses, and animals. The intra- and inter-specific divergences were calculated based on different taxa. Sequences were aligned using Clustal W, and Kimura 2-parameter (K2P) distances were calculated using PAUP4b10 (Florida State University, USA). The intra-specific variations and inter-specific divergences of congeneric species in the dicotyledons, monocotyledons, gymnosperms, ferns, mosses, and animals were calculated using a K2P distance matrix, as described previously [11], [31], [32].

### Species Identification

All ITS2 sequences of plants and animals were used as query sequences. Query sequences were divided into the following: dicotyledon, monocotyledon, gymnosperm, fern, moss, and animal. BLAST1, which was implemented using the BLAST program (Version 2.2.17), was used to search for the reference database for each query sequence [33].

### Secondary Structure of the ITS2 Region

To identify the effect of primary sequence divergences on secondary structure, ITS2 sequences with different sequence divergence (∼1%, ∼5%, ∼10%) were subjected to the secondary structure prediction in a genus that had three other species and three other genera in the same family. *Paphiopedilum* (Orchidaceae) of monocotyledons, *Acaena* (Rosaceae) of dicotyledons, and *Heterodera* (Ceratopogonidae) of animals were used to construct secondary structures using tools from the ITS2 database [35].

### Web Application for ITS2-based Species Determination

We developed a web application (http://its2-plantidit.dnsalias.org) to facilitate the utilization of the ITS2 sequence for various DNA barcoding studies. DNA sequences related to ITS2 regions were retrieved from GenBank, and were preprocessed to remove the flanking 5.8S and 28S rRNA gene sequences, as described in section Reference Database Construction. Sequences that belong to the same species, indicated by having the same taxonomy ID, were assembled using the program Phrap. The consensus sequence of the corresponding sequence clusters was considered as the average or reference sequence of the ITS2 region for the species, which can be retrieved from the application. The web application was built using the Catalyst web application framework (http://www.catalystframework.org/) for Perl language running in a Fedora 12 environment. This web application consists of four analytic modules at the time of the writing: View, Retrieve, Annotate, and Identify.

## Supporting Information

Table S1 No. of genera, species, and samples used in this study.
(0.03 MB DOC)

Table S2 Success rates of using ITS2 sequences to identify dicotyledon, moss, and gymnosperm species in families having less than 10 genera and monocotyledon species in families having less than 5 genera.
(0.05 MB XLS)

Table S3 Success rates of using ITS2 sequences to identify dicotyledon species in genera having less than 80 species, monocotyledon species in genera having less than 30 species, gymnosperm, moss, and fern species in different genera and animal species in genera having less than 20 species.
(0.39 MB XLS)

Table S4 Sequences that may be of fungal origin.
(0.03 MB XLS)

Table S5 The sequences of the universal primers and the amplification conditions for obtaining the ITS2 sequences of plants and animals.
(0.03 MB DOC)

Table S6 Samples used to determine the potential for using ITS2 sequences to identify species, and their accession numbers in GenBank.
(5.91 MB XLS)

Figure S1 Alignment of primary sequences of dicotyledons. (A) Alignment of the primary sequences of four species from the genus Acaena of Rosaceae; (B) Alignment of the primary sequences of four species from four genera of Rosaceae; and (C) Alignment of the primary sequences of four species from four families of dicotyledons.
(0.03 MB PDF)

Figure S2 Secondary structure of ITS2 in different species of monocotyledons.
(4.00 MB TIF)

Figure S3 Alignment of the primary sequences of monocotyledons. (A) Alignment of the primary sequences of four species from the genus Paphiopedilum of Orchidaceae; (B) Alignment of the primary sequences of four species from four genera of Orchidaceae; and (C) Alignment of the primary sequences of four species from four families of monocotyledons.
(0.03 MB PDF)

Figure S4 Secondary structure of ITS2 in different species of animals.
(3.86 MB TIF)

Figure S5 Alignment of the primary sequences of animals. (A) Alignment of the primary sequences of four species from the genus Heterodera of Heteroderidae; (B) Alignment of the primary sequences of four species from four genera of Heteroderidae; and (C) Alignment of the primary sequences of four species from four families of animals aided by secondary structure using 4SALE [47].
(0.04 MB PDF)
