# Supplementary material for: Use of ITS2 Region as the Universal DNA Barcode for Plants and Animals
Source: PLoS One. 2010 Oct 1;5(10):e13102. doi: 10.1371/journal.pone.0013102 (PMC2948509; doi:10.1371/journal.pone.0013102)
Supplement: Table S1 — No. of genera, species, and samples used in this study. (0.03 MB DOC) [file pone.0013102.s001.doc]

Table S1. No. of genera, species and samples used in this study.

| Taxa | No. of families | No. of genera | No. of species | No. of samples |
| --- | --- | --- | --- | --- |
| Animals | 283 | 516 | 3188 | 12221 |
| Dicotyledons | 159 | 1663 | 19133 | 34676 |
| Monocotyledons | 35 | 572 | 6105 | 11598 |
| Gymnosperms | 9 | 39 | 439 | 946 |
| Mosses | 56 | 171 | 1268 | 3528 |
| Ferns | 2 | 2 | 10 | 42 |
