# Supplementary material for: Use of ITS2 Region as the Universal DNA Barcode for Plants and Animals
Source: PLoS One. 2010 Oct 1;5(10):e13102. doi: 10.1371/journal.pone.0013102 (PMC2948509; doi:10.1371/journal.pone.0013102)
Supplement: Table S5 — The sequences of the universal primers and the amplification conditions for obtaining the ITS2 sequences of plants and animals. (0.03 MB DOC) [file pone.0013102.s005.doc]

Table S5. The universal primers and amplification conditions for obtaining the ITS2 sequence of plants and animals

(1) The sequences of the universal primers for PCR amplification of ITS2 sequences of plants have been described in Chen et al. (2010) and are shown below.

ITS2F: 5'- ATGCGATACTTGGTGTGAAT-3'

ITS3R: 5'- GACGCTTCTCCAGACTACAAT-3'

Thermal Cycler program:

94 °C 5 min

94 °C 30 s, 56 °C 30 s, 72 °C 45 s, 40 cycles

72 °C 10 min

(2) The sequences of universal primers for PCR amplification of ITS2 sequences of animals have been described in White et al. (1990) and are shown below.

ITS3: 5'- GCATCGATGAAGAACGCAGC-3'

ITS4: 5'- TCCTCCGCTTATTGATATGC-3'

Thermal Cycler program:

95 °C 2–3 min

94 °C 30 s, 50–60 °C 30 s, 72 °C 30–120 s (depending on product size), 25 cycles

72 °C 10 min

**References**

Chen S, Yao H, Han J, et al. 2010. Validation of the ITS2 region as a novel DNA barcode for identifying medicinal plant species. PLoS ONE, 5(1): e8613. doi:10.1371/journal.pone.0008613

White TJ, Bruns T, Lee S, Taylor J. 1990. Amplification and direct sequencing of fungal ribosomal RNA genes for phylogenetics. In: Innis M, Gelfand D, Swinsky J, White TJ, eds. PCR protocols: a guide to methods and applications. San Diego, CA: Academic Press, 315–322.
