# Supplementary material for: Use of ITS2 Region as the Universal DNA Barcode for Plants and Animals
Source: PLoS One. 2010 Oct 1;5(10):e13102. doi: 10.1371/journal.pone.0013102 (PMC2948509; doi:10.1371/journal.pone.0013102)
Supplement: Figure S1 — Alignment of primary sequences of dicotyledons. (A) Alignment of the primary sequences of four species from the genus Acaena of Rosaceae; (B) Alignment of the primary sequences of four species from four genera of Rosaceae; and (C) Alignment of the primary sequences of four species from four families of dicotyledons. (0.03 MB PDF) [file pone.0013102.s007.pdf]

Figure S1. Alignment of the primary sequences of dicotyledons. Identical sequences are indicated by (*).

(A) Alignment of the primary sequences of four different species from the genus *Acaena* of Rosaceae.

```
AY634855        ACACGTCTTTGCCACCGCAACCCCTTCGGGGGTTGGAAGGGATGGATGATGGCCTCCCGT     60
AY634796        ACACGTCTTTGCCACCGTAACCCCTCCGGGGGTTGGAAGAGATGGATGATGGCCTCCCGT
AY634784        ACACGTCTTTGCCACCGCAACCCCTTCGGGGGTTAGAAGGGATGGATGATGGCCTCCCGT
AY634831        ACACGTCTTTGCCACCGCAACCCCTTCGGGGGTTGGAAGGGATGGATGATGGCCTCCCGT
                ***************** ******* ******** **** ********************

AY634855        GTGCTCCGTCTCGTGGCTGGCATAAGTACCAAGTCCCAGAAAATCGGTGGTTATGAGACC     120
AY634796        GTGCTCCATCACGTGGCTGGCATAAGTACCAAGTCCCTGAAAATCGGTGGTTATGAGACC
AY634784        GTGCTCCGTCACGCGGCTGGCATAAATACCAAGTCCCCAACAATCGGTGGTTATGAGACC
AY634831        GTGCTCTGTCACGCGGCTGGCATAAATACCAAGTCCCCGACAATCGGTGGTTATGAGACC
                ******  ** ** *********** ***********  * *******************

AY634855        TCGGTGTCCGGTCGTGCGCGCGCATCTTTTGTTGGCCTTCATGATGCGCGTTGATCCGTC     180
AY634796        TCGGTGTCCGGTCGTGCGCGCGCGTCTTTTGTTGGCCTTCATGATGCGCGTTGATCCGTC
AY634784        TCAGTGTCCGGTCGTGCGCGCGCGCCTGTTGTTGGCCTTCATGATGTGCGTTGATCCGTC
AY634831        TCAGTGTCCGGTCGTGCGCGCGCGTCTGTTGTTGGCCTTCATGATGTGCGTTGATCCATC
                ** ********************  ** ****************** ********** **

AY634855        AACACTTTCAACGC     194
AY634796        AACACTTTCAACGC
AY634784        AATGCTTTCAACGT
AY634831        ATCGCTTTCAACGT
                *   *********
```

(B) Alignment of the primary sequences of four species from different genera of Rosaceae.

```
AY634796    ACACGTCTTTGCCACCGTAACC-CCTCCGGGGGTTGGAAGAGATGGATGATGGCCT-CCC    60
AF163482    ACACGTCGTTGCCCCCCCGACC-CCTTCGGGGGTCGGACGGGACGGATGATGGCCTTCCC
EF050800    ACACGTCGTTGCCCCCCCAACCTCCCTCGGGAGTTGGGCGGGACGGATGATGGCCT-CCC
AF411508    ACACGTCGTTGCACCCCCACTACTCCCTCGGGATTGCGGGGTGCGGATGATGGCCT-CCC
            ******* ****  **        *    **  * *   *    ************ ***

AY634796    GTGTGCTCCATCACGTGGCTGGCATAAGTACCAAGTCCCTGAAAA---------------    120
AF163482    GTGTGCCCCGTCACGCGGTTGGCATAAATACCGAGTCCTCGGCGACCGGCGYCGCGGCGA
EF050800    GTCTGCTCTGTCATGCGGTTGGCATAAAAAC-AAGTCCTCGGCGACTAACGCCACGACAA
AF411508    GTACGCTCCGTCGTGCGGTTGGCATAAATACCAAGTCCTCGGCGACGCACGCCACGACAA
            **  ** *  **  * ** ********  **  *****  *   *

AY634796    TCGGTGGTTATGAGA-CCTCGGTGTCCGGTCGTGCGCGCGCGTCTTTTGTTGGCCTTCAT    180
AF163482    TCGGTGGTTGTCAAA-CCTCGGTGCCTTGTCGCGTGCGTGAGTCGATCGCGGGACTTCCT
EF050800    TCGGTGGTTTCCAAAACCTCTGTTGCCTGTCGTGTTCGCGTGTCGGACGAGGG-CTCCCC
AF411508    TCGGTGGTTGCGAAA-CCTCGGTTGCCCGTCGTGTGCGGTCGTCGCGCATCGGGGGCTCG
            *********   * * **** **  *  **** *  **   ***       **

AY634796    GAT------GCGCGTTGATCCGTCAACACTTTCAACGC    219
AF163482    TAGCCGTGAGCGCGTCGGTAACCCGACGCTTTCAACGC
EF050800    AAACCATG-TTGCGTCGATTCGTCGATGCTTTCAACG-
AF411508    AAA----AAATGCTTGGCTCCGGCTTGGCTTTCAACGC
             *         ** * * *    *   *********
```

(C) Alignment of the primary sequences of four species from four families of dicotyledons.

```
EF538294        AACACGTCACCTCCCAGCACACCTCTTGATGGGGATGTTGTCGTGGGGGCGGAGATTGGT    60
EF050800        -ACACGTCGTTGCCCCCCCAACCTCCC--TCGGGA-GTTG--GGCGGGACGGATGATGGC
AY996481        ATCGCGTCGCC-CCCCGCACGCCGCTC---------GGCGTCGTGGGGGCGGATACTGGC
AB051978        -ACATATCGTTGCCCGATGCCTATTGCAGTGCAGTAGGAATTTCTAGGGCGAATGATGGC
                  *   **    ***                     *         ** ** *   ***

EF538294        CTCCCGTTC---CTAAGGT---GCGGTTGGCTAAAATAGGAGTCCCCGACGAAGGACGC-    120
EF050800        CTCCCGTCTGCTCTGTCAT---GCGGTTGGCATAAAAACAAGTCCTCGGCGACTAACGCC
AY996481        CTCCCGTGCGCCTCGCGCCC--GCGGCCGGCCTAAATGCGAGTCCACGTCGACGGACGTC
AB051978        TTCCCGTGAGCGTTGTTGCCTCGCGGTTGGTTGAAAATCGAGTCCTTGGTAGGGTGTGCC
                 ******               ****  **   ***    *****  *         *

EF538294        ACGCTTAGTGGTGGTTG--ACATGACCCTCTTAT---CGAATCGTGTGTTCAAAGGAGGG    180
EF050800        ACGACAATCGGTGGTTT--CCAAAACCTCTGTTG---CCTGTCGTGTTCGCGTGTCGGAC
AY996481        GCGGCAAGTGGTGGTTGTAACCCAACTCTCTTGGTGCCGCGGCTCATGCCCGTCGCTCGT
AB051978        ATGGTAGATGGTGGTCGAGTTAGAACGATACCGA---TCATGTGCATGCTCCCCA-AAGT
                  *      ******         **                    *   *

EF538294        AAGATCTCTTTGATGACCCTAATGTGTTGTCTTGTACGATGCTTCGACC-    230
EF050800        GAGGGCTCCCCAA--ACCATGTTGCGTCGATTCGT-CGATGCTTTCAACG
AY996481        GCGTGCTCCCCGA--CCCTTTAGGCG----CTCGC---GCGCTCCGACC-
AB051978        ATGGCCTCTATGA---CCCATAAGCG-----TCTTTTGGCGCTCATGA--
                  *  ***    *   **     * *     *        ***
```
