# Supplementary figures and images for: Use of ITS2 Region as the Universal DNA Barcode for Plants and Animals
Source: PLoS One. 2010 Oct 1;5(10):e13102. doi: 10.1371/journal.pone.0013102 (PMC2948509; doi:10.1371/journal.pone.0013102)

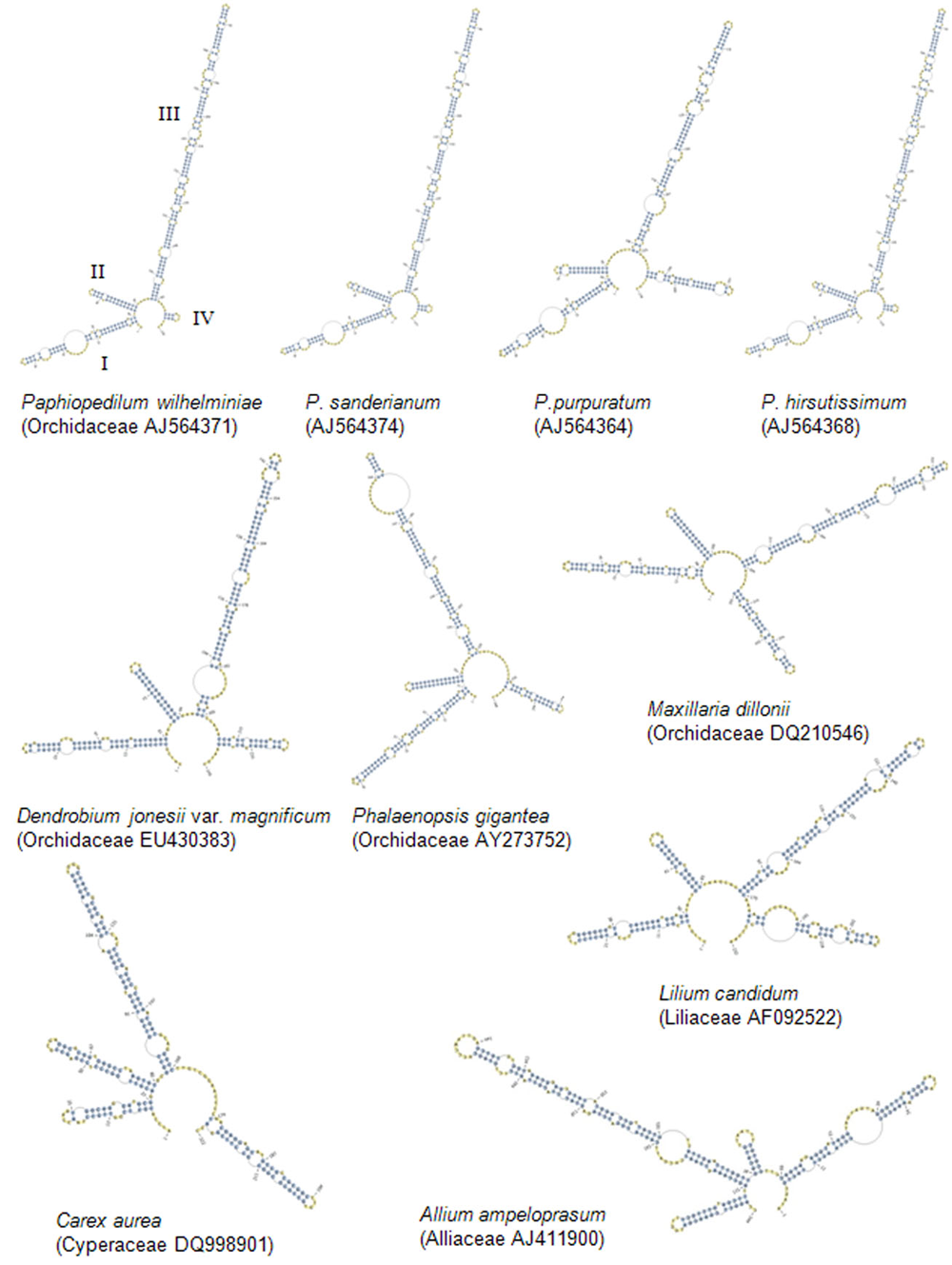

Supplement: Figure S2 — Secondary structure of ITS2 in different species of monocotyledons. (4.00 MB TIF) [file pone.0013102.s008.tif]

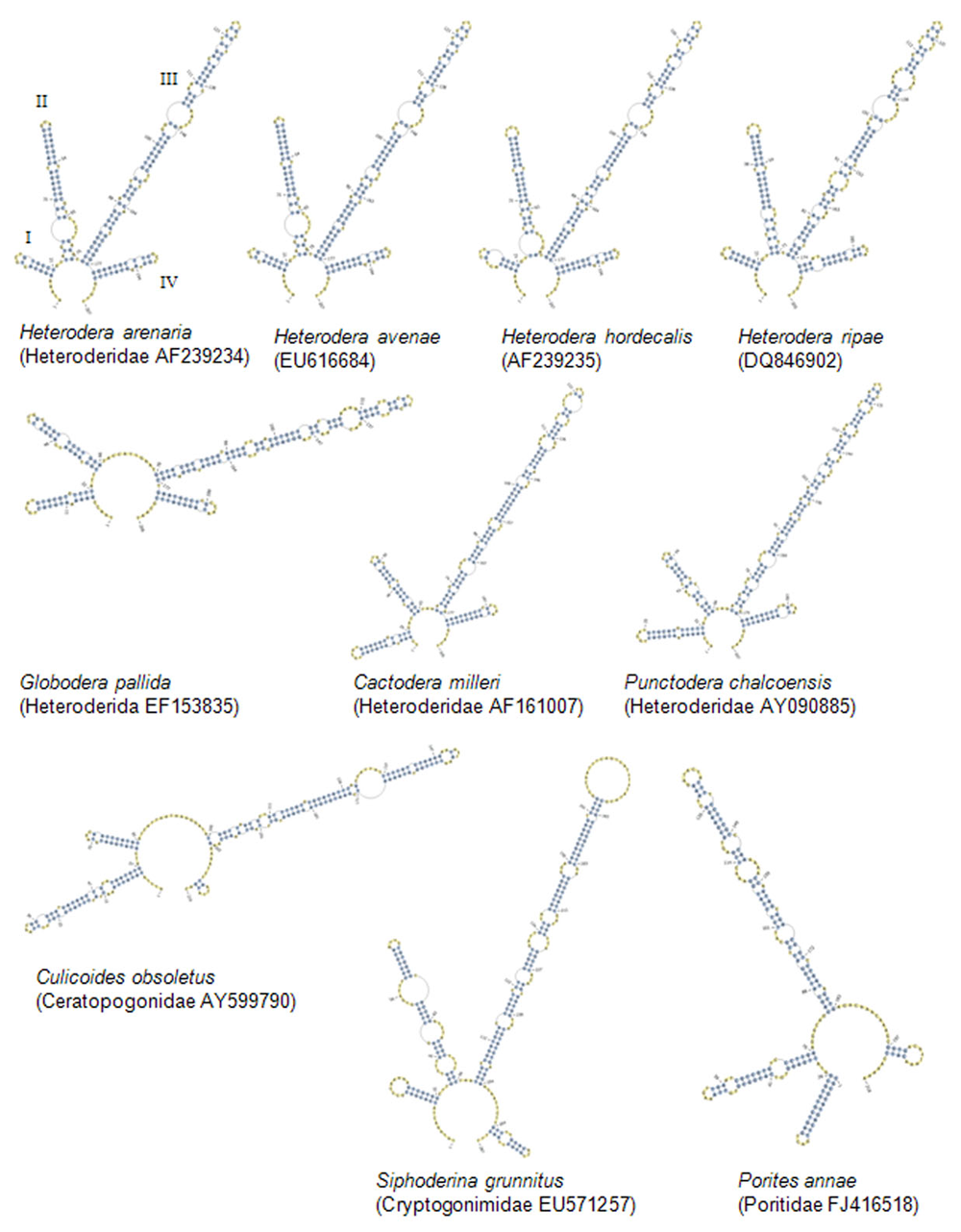

Supplement: Figure S4 — Secondary structure of ITS2 in different species of animals. (3.86 MB TIF) [file pone.0013102.s010.tif]
