# Supplementary material for: Use of ITS2 Region as the Universal DNA Barcode for Plants and Animals
Source: PLoS One. 2010 Oct 1;5(10):e13102. doi: 10.1371/journal.pone.0013102 (PMC2948509; doi:10.1371/journal.pone.0013102)
Supplement: Figure S3 — Alignment of the primary sequences of monocotyledons. (A) Alignment of the primary sequences of four species from the genus Paphiopedilum of Orchidaceae; (B) Alignment of the primary sequences of four species from four genera of Orchidaceae; and (C) Alignment of the primary sequences of four species from four families of monocotyledons. (0.03 MB PDF) [file pone.0013102.s009.pdf]

Figure S3. Alignment of the primary sequences of monocotyledons. Identical sequences are indicated by (*).

(A) Alignment of the primary sequences of four different species from the genus *Paphiopedilum* of Orchidaceae.

```
AJ564371        GCGAGTCATATCTCTCCCTTAATGAGGCTGTCCATACATACTGTTCAGCCGGTGCGGATG    60
AJ564364        GCGAGTCATATCTCTCCCTTAACGAGGCTGTCCATACATACTGTTCAGCCGGTGCGGATG
AJ564374        GCGAGTCACATCTCTCCCTTAATGAGGCTGTCCATACATACTGTTCAGTCGGTGCGGATG
AJ564368        GCGAGTCATATCTCTCCCTTAATGAGGCTGTCCATACATATTGTTCAGTCGGTGCGGATG
                ******** ************* ***************** ******* ***********

AJ564371        TGAGTTTGGCCCCTTGTTCTTTGGGACGGGGGGTCTAAGAGCTGCATGGGCTTTTGATGG    120
AJ564364        TGAGTTTGGCCCCTTGTTCTTTGCTACGGGGGGTCTAAGAGCTGCATGGGCTTTTGATGG
AJ564374        TGAGTTTGGCCCCTTGTTCTTCGGTACGGGGGGTCTAAGAGCTGCATGGGCTTTTGATGG
AJ564368        TGAGTTCGGCCCCCTGTTCTTCGGTACGGGGGGTCTAAGAGCAGCATGGGCTATCGATGG
                ****** ****** ******* *  ***************** ********* * *****

AJ564371        TCCTAAATACGGCAAGAGGTGGACGAACTATGCTGCAACAAAATTGCTGTGCGAATGCCC    180
AJ564364        TCCTAAATACGGCAAGAGGTGGACGAACTATGCTACAGCAAAATTGTTGTGCAAAGGCCC
AJ564374        TC-TAAATACGGCAAGAGGTGGACGAACTATGCTACAACAAAATTGTTGTGCGAATGCCC
AJ564368        TCCTAAATACGGCAAGAGGTGGACGAACTATGCTACAACAAAACTGTTGTGCGAATGCCC
                ** ******************************* ** ***** ** ***** ** ****

AJ564371        CGGGTTGTCGTATTAGATGGGCCAGCATAATCTAAAGACCCTTTTGAACCCCATT-GGAG    240
AJ564364        CGGGTTGTCGTATTAGATGGGCCACCGTAGTCTGAAGACCCTTTTGAACCCCATT-GGAG
AJ564374        CGGGTTGTCGTATTAGATGGGCCAGCATAATCTAAAGACCCTTTTGAACCCCATT-GGAG
AJ564368        CGGGTTGTCGTATTAGATGGGCCAGCGTAATCTAAAGACCCTTTTGAACCCCATTTAGAG
                ************************ * ** *** *********************  ***

AJ564371        GCCCATCAACCCATGATCAGTTGACGACCATTTGGTTGC    279
AJ564364        GCCCATCAACCCATGATCAGTTGATGGCCATTTGGTTGC
AJ564374        GCCCATCAACCCATGATCAGTTGACGGCCATTTGGTTGC
AJ564368        GCCCATCAACCCATGATCAGTTGGGGGCCACATGGTTGC
                ***********************  * ***  *******
```

(B) Alignment of the primary sequences of four species from different genera of Orchidaceae.

```
EU430383        --------CATCGCTCCGTGCCGA--CTCCGTCCCATC---AATGGGTGGGCCGGCGGAG    60
DQ210546        AAGCGTCGCGTCGCTCCGTGCCAC--CTGCGTCCCGCC---AGCGGGC--GTCGGTCGAG
AY273752        AAGCGTTGCGCCGCTCCGTGCCGAGCCCCCATCCCCGCCGCGGTGGGGGTGCCGGGCGAG
AJ564368        --GCGAGTCATATCTCTCCCTTAATGAGGCTGTCCAT-----ACATATTGTTCAGTCGGT
                        *    ***             *   **                 * *  *

EU430383        GCTCGGATGTGGAGAGTGGCTCGTCGTGCCCATCGGTGCGGCGGGCTGAAGAGCGGGTGA    120
DQ210546        GCCCGGATGTGCAGAGTGGCTCGTCGTGCCCGTCGGCGCGGCGGGCTGAAGAGCGGGTT-
AY273752        GCCCGGATGTGCAGGGTGGCCCGTCGTGCCCATCGGCGCGGCGGGCTGAAGAGCGGGTTA
AJ564368        GC--GGATGTG-AGTTCGGCCC--CCTGTTCTTCGGTACGGGGGGTCTAAGAGCAGCATG
                **  ******* **   *** *  * **  * ****  *** ***   ****** *

EU430383        TCATCTCGTTGGCCGCGAACA--ACAAGGGGTGGATGAAAG-------C---GAGGCCTA    180
DQ210546        TCGTCTCGCCGGCCGCGAACA--ACAAGGGGTGGGTGAAAG-------CTGTGAGCGCAG
AY273752        TCGTCTCATTGGCCACGAACA--ACGAGGGGTGGATGAAAGAAAGCTGCCGCGGGCAAGG
AJ564368        GGCTATCGATGGTCCTAAATACGGCAAGAGGTGGACGAACTAT-GCTACAACAAAACTGT
                   * **   ** *   ** *   * ** *****  ***         *

EU430383        CGT-TGT-TGTGTCGTGTTTGCCGGAGAGA-GGATT---GCACTT------CTCAGGTGA    240
DQ210546        CCTGCGT-TGTCTCGTGCCGGCCCGAGAGACGGGCC---GTGCCT------ACCATGTGA
AY273752        CCCGCGT-TGTCTCGTGCCGGCCGGAGAGG-AGATT---TCACCC------TTCGTGCGA
AJ564368        TGTGCGAATGCCCCGGGTTGTCGTATTAGATGGGCCAGCGTAATCTAAAGACCCTTTTGA
                     *  **   ** *    *     **   *                    *    **

EU430383        TCCCAAACCATGCGTCGATCCACGGACGGCGGCTTGGAATGC----------    292
DQ210546        TCCCGGCCCATGCGCCGATCGGCATGCGGCGGCTTGGAATGC----------
AY273752        TCCCATCCCACGCGCCGCCCC-CGTGCGGCGGCTTGGAATGC----------
AJ564368        ACCCCATTTAGAGGCCCATCAACCCATGATCAGTTGGGGGCCACATGGTTGC
                 ***     *   * *   *  *    *     ****    *
```

(C) Alignment of the primary sequences of four species from four families of monocotyledons.

```
DQ998901    -------------ATCCACGCTCG-GTTGCCTAACCAATGG-CAA------------GGA    60
AF092522    ----CTTGTTTCGCTCTATGCCCATGCTCCTTCGGGGGCGGTCAT------------GGA
DQ210546    AAGCGTCGCGTCGCTCCGTGCCACCTGCGTCCCGCCAGCGGGCGTCGGTCGAGGCCCGGA
AJ411900    ----ATAGCGTCATTCCAATCCCCCAAAATGCGATGAGTGCAGTTTGGGATGATGATGGA
                          **    *                  *                 ***

DQ998901    TGCGGACGTTGGCCCTCCGAGCCGCG-AGGCGCGGTGGGCCTAAGTGTGCGGCCGTCG-T    120
AF092522    TGCAGATATTGGCCCTCCGTGCCTCG-TG-TGCGGCGGGCTTAAGCGCG-GGCTGTCGGC
DQ210546    TGTGCAGAGTGGCTCGTCGTGCCCGT-CGGCGCGGCGGGCTGAAGAGCG-GGTTTCGTCT
AJ411900    TATGGAGAATGACCTTCCGTGCTTTAATTGTACGGTTGGTTTAAGTT----ACTGTCATT
            *    *   ** *    ** **          ***  **   ***

DQ998901    GCGTGG-CCGGGAGCGGCGAGTGGTGGGCT-A--CTGCGCACGTCACCC-CGAGCCCCGT    180
AF092522    GTCGGG-ATGGACACGACGAGTGGTGGACGGA--GCACCAGTAGGATGT-CGTGGTCCCC
DQ210546    CGCCGG-CCGCGAACAACAAGGGGTGGGTGAAAGCTGTGAGCGCAGCCTGCGTTGTCTCG
AJ411900    GCCAGGTATATGCGAGGCAAATGGTGTGTCGAGTTAACCCACGATGTCTCTAATTGCATC
                **           * *  ****     *                        *

DQ998901    AACGATGCAGGGCCTTGTCGGAACCCCTAACGAGGAGCATGCCGCCG-CG-GCCTGT-GC    240
AF092522    CGTCGCCTAAAGGGGCTCAAAAAATCCGGACTAGGCGAGCCGTGCCC-CGTACGAGG-AG
DQ210546    TGCCGGCCCGAGAGACG--GGCCGTGCCTACCATGTGATCCCGGCCCATGCGCCGATCGG
AJ411900    CATGAGATCTAGGCATG-----ACTTAGCACTAATTGAAACCGATTTCGATGTTTGC--T
                       *                 ** *   *

DQ998901    TGCGCGGCGCCTTCGGACC-    260
AF092522    GGCGAGCTGTCTCCCAA---
DQ210546    CATGCGGCGGCTTGGAATGC
AJ411900    TTGGTAGCAAGCTCGGACC-
               *            *
```
