# Supplementary material for: Use of ITS2 Region as the Universal DNA Barcode for Plants and Animals
Source: PLoS One. 2010 Oct 1;5(10):e13102. doi: 10.1371/journal.pone.0013102 (PMC2948509; doi:10.1371/journal.pone.0013102)
Supplement: Figure S5 — Alignment of the primary sequences of animals. (A) Alignment of the primary sequences of four species from the genus Heterodera of Heteroderidae; (B) Alignment of the primary sequences of four species from four genera of Heteroderidae; and (C) Alignment of the primary sequences of four species from four families of animals aided by secondary structure using 4SALE [47]. (0.04 MB PDF) [file pone.0013102.s011.pdf]

Figure S5. Alignment of the primary sequences of animals. Identical sequences are indicated by (*).

(A) Alignment of the primary sequences of four different species from the genus *Heterodera* of Heteroderidae.

```
AF239234      ATAAAAGGCACTGCTGTGCCTGTTATGTTGGTGAGATCATGTCGGCATGACGTGTTCTTG    60
EU616684      ATAAAAGGCACTGCTGTGCCTGTTATGTTGGTGAGATCATGTCGGCTTGACGTGTTCTTG
AF239235      ATAAAAGGCACTGCTTTGCCTGTTATGTTGGTGGGATCATGTCTTCTTGACGTGTTCCTG
DQ846902      ATAAAATGCACAGCTGTGCGTGTTGATCTCGTGGGATCATGTCTGTTTGACGTGTTCTTG
              ****** **** *** *** ****    * *** *********    ********** **

AF239234      CGCTATTCTTGAAAATGCTCGGCCGTGGAGTGTGGTTGTGTTGGCGCGAAACTGTCAGGT    120
EU616684      CGCTATTCTTAAAAATGCTCGGCCGTGGAGTGTGGTTGTGTTGGCGCGATACTGTCAGGT
AF239235      CGCTGTTTGTTCAA-TGCCCGGCTGTGGGATGTGGTTGTGTTGGCGCGAAACTGTCAGGT
DQ846902      CGTTATGTTTG----TGCCCGGCTATGGGATTTGGTTATGCTAGCGCGAAACTGTCAGTT
              ** * *   *     *** ****  ***  * ***** ** * ****** ******** *

AF239234      T-CTTTCGCGTTTTACGGTCCGTAACTAGGACGCAACTGCTCGCCATGTGTGCTG-GGTG    180
EU616684      T-CTTTCGCGTTTTACGGTCCGTAACTAGGACGCAACTGCTCGCCTTGTGTGCTG-GGTG
AF239235      TATTTTCGCGTTTTACGGTCCGTAACTCGGACACAACTGCGTTCCATGTATGCTG-GGCG
DQ846902      T-ATTTCGCGTTTTACACACCGTAACTCGGGCGTGGCCATTTCTCATGAATGCTGTGGTG
              *  *************   ******** ** *    *       * **  ***** ** *

AF239234      GAAT——GCTTCGCTGGTAGGCATTCCGTGTT    211
EU616684      GAAT--GCTTCGCTGGTAGGCATTCCGTGTT
AF239235      GAGT--GCTTCGCTGGTAGGCATTCCATGTT
DQ846902      GAATATGCCTAGCATGT-GGCGTTCCGTGTT
              ** *  ** * **  ** *** **** ****
```

(B) Alignment of the primary sequences of four species from different genera of Heteroderidae.

```
EF153835        ---CAAAAAATGCACTGCATGTGCGTGTTTTATTTGCTAA-GATCACGC---TTCGGCGT    60
AY090885        ACCAAAAAAAATGCACTGCATGTGCATGTTTTTGTTGCTAGTAATCACGC---TCCGGCGT
AF161007        --CCAAATAATGCACTGCATGTGCGTGTTTTGACTGCTAA-GATCACGC---CTGGTCGT
DQ846902        ----ATAAAATGCACAGC-TGTGCGTGTTGATCTCGTGGG--ATCATGTCTGTTTGACGT
                    * * ******* ** ***** ****      *      **** *       * ***

EF153835        GTTCTTGCATACCATTGAATGCTACGCTGTGT--AGTGTTGGACGTGCTGGCGCGAAAAT    120
AY090885        GTTCTTGCATAACATTGATTG-TACGCTGTGTGTAGCGTGGGAGGGTGTGGCGCGAGAAT
AF161007        GTTCTTGCATAAAACTATGCG-TACGCTGTGT--GGCGTTGGACGTGCTGGTGCGGAAAT
DQ846902        GTTCTTGCGTTATGTT-TGTGCCCGGCTATGG---GATTTGGTTATGCTAGCGCGAAACT
                ******** *     *    *    *** **    *  * **      * * ***  * *

EF153835        GTGTTGT---CTTTCGCGCTTTACAGACCGTAATTTAGGCACGCCCTTCGTTCAC--ATG    180
AY090885        GTGTTGT---ATTTCGCGCTTTACAGACCGTAATTCAGGCGCCCCTCTCGTTCAC--ATG
AF161007        GTGTTGTTCTCTTCCGTGCTTTACAGACCGTAATTTAGGTACGTCCCTCGTTCAT--ATG
DQ846902        GTCAGTT--TATTTCGCGTTTTACACACCGTAACTCGGGCGTGGCCATTTCTCATGAATG
                **    *    ** ** * ****** ******* *  **     *  *   ***   ***

EF153835        CGATAGCTGAATGC-CTCGCCAATAGGCATTCGCAATTGAACATTTCGACCTGAA    235
AY090885        TGATAGCGGAATGC-CTCGACAATAGGCATTTGCAAC------------------
AF161007        CGATAGCTAAATGC-CTCGCCAATAGGCATTTGTACT------------------
DQ846902        CTGTGGTGGAATATGCCTAGCATGTGGCGTTCCGTGTT-----------------
                   * *   ***   *    **   *** **
```

(C) Alignment of the primary sequences of four species from four families of animals.

```
>AY090885           -------------ACCA-AAAAAATG-C-AC--TGCATGTGCATGT-TTTTG-T-TGCT-   60
>FJ416518           ---------------CG-CGC--ATGAC-TT--GAC-TGAGT-CGT-GTGCGGTAT--TG
>EU571257           -------TAT-AAACTA-T-C--ACG---AC--GCC-CAAAA-AGTCGTG-GCT----T-
>AY599790           ATTTATATATTAAACTATCTT--ATG-TGTCTTTAA-CGGGC-ATC-ATAAG-T----GC
                                           * *                      *  * *

>AY090885           A-G---TA-AT--CA-C-G-C-TC-------CG--GCG-TG-TTC-TTGC-ATAAC-AT-   120
>FJ416518           A-G--GTG--T--CA-C-GGCGTG----TTAAA--GT--TGAATCGCCGT-GTCCC--T-
>EU571257           G-G--GTC-TTGCCAGCTGGCGTGATTTCCCTGCAATTCTG-TGGGGTGCCGGATCTATG
>AY599790           ATGATGTGATCA-TA-T-GGA-TG-------TA--AC--AA-TTC-ATAT-ATCA---T-
                      *   *       *   *   *                                   *

>AY090885           --TGATTG-TA-C-G---CTGTGTGTAG--CG--T-----G-G------G---AGG-GTG   180
>FJ416518           --TGAAGGGCAGCAG---C-AT-TGGACT-CGCATTCTCTATG------G---AAACTTG
>EU571257           GCTTTTCCCTAAT-GTGCC-GGATGCACC-CACTC-CT-GG-GCTGATTGCTTAGGTGAG
>AY599790           --TAAT---AA-A-G---G-ATATGGTTAACGC-TACT-TT-G------T---GTGCATA
                      *       *   *         **    *           *

>AY090885           -T--GG-CG-CG-A---G-A-----AT----GT-GT-TGTATTT--CG-CGC-TT-TACA   240
>FJ416518           ----AG-TG-CG-AA--G-G-----CTAA-AG--AT-ACTATTTTCCTTCGC-CC-AA-A
>EU571257           GTGGAGACGGCGGAGTCGTGGCTCAATTTCAGTCATATATATACG-CA-CGCTCCGTTGT
>AY599790           TTT-AACCA-TT-AT-TTTT-----AT-------CT-AAAAAAT--AA-TAAATT-TTGG
                                 *            *        *    *

>AY090885           -GACCGT-AATTCAGGC---G-CCC-CTC-TCGTTCACATGTG-AT-A-GCGGAATGCCT   300
>FJ416518           -AAACCA-ATAGCAGAC---GGTCC-CAA-TAGCTGAGACAAACAA-A-GCGG-------
>EU571257           CGAGCCTCATTGATGGCTTAGGCTTACAAGTAGTGGTGATGTATCTGATGCAA-------
>AY599790           -TATAAA-ATCAAATGT---G-TAG-CAA-ATGTT-T-A-GT--AA-A-CCAA-------
                      *     *           *     *     *     *        *  *

>AY090885           CGA--CAA---TA----GGCAT-TT—GCAAC    332
>FJ416518           TGA--CAA---TA----AACAT-CC--AAAAA
>EU571257           CAATTCTTGTTTTGCACATTGTGCTTAACTTT
>AY599790           -AA---AA---AT----CTTAA-TT--ATGAC
                      *
```
